# The reliability and validity of the isixhosa version of the euroqol toddler and infant populations (EQ-TIPS) health related quality of life instrument

**DOI:** 10.4314/ahs.v23i4.63

**Published:** 2023-12

**Authors:** Janine Verstraete, Razia Amien

**Affiliations:** 1 Department of Child and Adolescent Health, Faculty of Health Sciences, University of Cape Town, Klipfontein Road, Rondebosch, Cape Town, South Africa; 2 Division of Physiotherapy, Department of Health and Rehabilitation Sciences, Faculty of Health Sciences, University of Cape Town, Anzio Road Observatory, Cape Town South Africa

**Keywords:** Child, infant, toddler, pre-schooler, health, health-related quality of life, HRQoL, proxy, outcome measure, isiXhosa, EQ-5D-Y

## Abstract

**Background:**

Considering the high burden of disease in young children measurement of Health-Related Quality of Life is needed to evaluate the burden of morbidity. This study aims to report on the validity and reliability of the isiXhosa EuroQol Toddler and Infant Populations (EQ-TIPS) measure for South Africa.

**Methods:**

A sample of 181 caregivers of children 0-36 months were recruited from a hospital in-patient (inpt) and outpatient (outpt) facility and crèches. The EQ-TIPS, Ages and Stages Questionnaire (ASQ), Faces, Leg, Activity, Cry, Consolability (FLACC) and dietary information were administered at baseline. EQ-TIPS was administered one week later in crèche children for test-retest reliability.

**Results:**

Known groups showed significant differences for pain (X^2^=37.21, p<0.001), and EQ-TIPS level sum score (KWH=25.9, p<0.001) between health groups. The Visual Analogue Scale was unable to discriminate general health between groups (KW-H=3.92, p=0.141). Concurrent validity was weak to moderate and significant for all dimensions hypothesised to correlate. There was significant fair to moderate test-retest reliability for EQ-TIPS dimensions of movement, play, pain and eating.

**Conclusion:**

The isiXhosa EQ-TIPS is valid and reliable for very young children in South Africa and we suggest that it be included in the assessment of children with health conditions within this context.

## Background

In Africa, children under five years of age comprise 16,5% of the total population 1. They also bear the highest burden of disease, with a mortality rate of 51% attributed primarily to poor nutrition, diarrheal disease, pneumonia, Tuberculosis (TB) and Human Immunodeficiency Virus (HIV) [1]. The World Health Organisation (WHO) launched the Sustainable Development Goals (SDGs), with a target date of 2030 in an effort to in part combat this issue 2. The third SDG aims to ensure healthy lives and promote well-being for all ages and includes areas of reproductive, maternal, new-born and child health 2. In response to this the ‘First 1000 Days’ initiative aims at further reducing the burden of maternal health and child mortality 3,4 The ‘First 1000 days’ of life is characterised from conception to two years of life. This is a vulnerable but instrumental period for optimal growth, brain development and health. This period lays the foundation for the child's life, however; this foundation is often weakened in Low and Low Middle-Income Countries by the determinants of poverty, environmental and societal hardships, ill-health and malnutrition 5,6. The programme is focused on nutritional support for both mother and child, access to quality health care, clean water and sanitation and social support systems. The initiative further aims to improve care and quality of life during this ‘First 1000 days’ period 4. There is an increasing awareness that in order to monitor health outcomes, both mortality and morbidity need to be assessed. Measurement of mortality is less complex and routine in most countries; however, the effects of morbidity are not often recorded. Morbidity results in considerable burden on health and social resources as well as a high burden of care for the family and society. Health Related Quality of Life (HRQoL) measures can assist in evaluating and monitoring the burden associated with morbidity and can therefore be useful measures to assess progress made in addressing initiatives such as the ‘First 1000 days.’ The EuroQol Toddler and Infant Populations (EQ-TIPS) (formerly the Toddler and Infant (TANDI) Health Related Quality of Life instrument) was recently developed in the South African context to measure HRQoL in this vulnerable childhood period of 0-36 months 7,8.

The EQ-TIPS was based on the EuroQol model of health status and aimed to be part of the EuroQol family of instruments to allow measurement and valuation of health across the lifespan. The EQ-TIPS includes six dimensions: movement, play, pain, relationships, communication and eating, with three levels of report (no problems, some problems, a lot of problems) 8. The measure has retained the EuroQol Visual Analogue Scale (EQ-VAS) 8,9 which captures perceived general health on a scale from 0 (worst health imaginable) – 100 (best health imaginable). The EuroQol Group currently considers the EQ-TIPS an experimental version which requires further development and testing. To date the content validity and psychometric properties of the EQ-TIPS have been tested in English and Afrikaans for South Africa 8,10–12 with a recommendation for further research to establish the clinical utility and cross-cultural validity in other languages and cultural contexts 8.

New language versions of outcome measures need to be cross-culturally adapted and show satisfactory psychometric properties before utilisation 13. Translation of the EQ-TIPS into African languages for South Africa is of great importance to not only increase the utility of the measure but to ensure that HRQoL can be accurately captured across all patient groups. isiXhosa, is the second most widely spoken African language in South Africa and the most widely spoken in the Western Cape Province 14 which was the setting for previous psychometric testing of the TIPS and this research. The aim of this study was thus to test the validity and reliability of the isiXhosa EQ-TIPS in young children and infants across the age range and different health conditions.

## Methods

A cross-sectional non-probability design was used to determine the validity of the EQ-TIPS. Longitudinal data was collected from the caregivers of a group of children attending crèche from the general population for test-retest reliability.

### Participants

The participants for this study included the caregivers of children aged 0-36 months drawn from in-patients and out-patients respectively at a children's hospital in Cape Town, South Africa. Participants also included caregivers from the general population attending several crèches and toddler play groups in Cape Town, South Africa (creche group). Caregivers had to be proficient in isiXhosa as this study aimed to determine the acceptability and validity of the isiXhosa version of the questionnaire. Caregivers of children who were admitted to the Intensive Care Unit, terminally ill or who were born prematurely and had not yet reached their corrected age of birth were excluded from the study. The sample size was considered for each psychometric property in accordance with the COSMIN guidelines where n>100 per group is considered very good for convergent validity and n=50-99 as adequate for reliability 15.

### Procedure

Ethical approval from the University Human Research and Ethics Committee (HREC 012_2019) and permission from the children's hospital and crèches was obtained. All caregivers of children attending the crèches were sent a detailed description of the study before data collection commenced. The research packs were given to the caregivers when they came to fetch their child from crèche and they were requested to return the envelope, sealed, with the completed research pack after three days. The research pack consisted of information regarding the study and informed consent, demographic and medical information, EQ-TIPS, The Ages and Stages Questionnaire (ASQ) Parent Report Form, Face, Leg, Activity, Cry, Consolability (FLACC) Pain Scale (for children aged 2-36 months) and Dietary Information. The order of the questionnaires was standardised for all participants. All instruments were self-complete and no caregiver training for completion of questionnaires was required. The caregivers who participated were requested to complete a second EQ-TIPS measure and return it to crèche in the sealed envelope after one week. The time period was selected as the health of the crèche children was not expected to change in this period and it ensured that care-givers did not remember what they answered on the first administration 16.

Caregivers of children were recruited systematically from the in patients wards of the children's hospital according to ward and cubicle number. Caregivers of children receiving out patients' health care were recruited from the waiting rooms of general and specialist clinics at the children's hospital. Caregivers completed the research pack in a private room or in the case of in patients, at their child's bedside if they preferred.

### Measures

#### EQ-TIPS

The EQ-TIPS was formerly known as the Toddler and Infant (TANDI) Health Related Quality of Life measure. The translation process of the EQ-TIPS into isiXhosa followed the EQ-5D-Y translation protocol 17. Forward translations from English to isiXhosa and from isiXhosa back to English were undertaken by experienced local translators who were fluent in both languages. Two translators independently forward and backward translated the measure, and they discussed all translations with the researcher to reach a consensus version. This consensus version was piloted through cognitive interviews to establish the conceptual equivalence of the translated concepts. The cognitive interviews were conducted with 10 caregivers of children aged between 0-36 months according to the EQ-5D-Y translation protocol.

The interview structure and questions were adapted from the EQ-5D-Y translation protocol. Participants were asked open-ended questions to determine the comprehensibility of the entire questionnaire, words they found difficult to understand (in the dimensions, severity levels, and instructions), and any suggested changes to make the questionnaire easier to understand. The participants were additionally asked what they understood from each question and severity level, and subsequently provide examples of children experiencing these problems. As no problems were reported during the cognitive interviews no changes were made to the consensus version.

The newly adapted isiXhosa version of the EQ-TIPS for South Africa was tested for validity. Problems across the six dimensions of the EQ-TIPS are described, similarly to the EQ-5D instruments 18, by a six-digit code. For example, the EQ-TIPS health state 111223 describes someone with no problems with movement, no problems with play, no problems with pain, some problems with relationships, some communication and a lot of problems with eating. The best health state described by the instrument is coded as 111111, describing ‘no problems’ in each of the dimensions. Thus, the EQ-TIPS has 729 (3^6^) unique health states. The EQ-TIPS does not have a preference-based score; therefore, a level sum score (LSS), similar to that used on the EQ-5D, was used to describe the responses on the descriptive system where the level labels are treated as numeric data with the best possible score (1 + 1 + 1 + 1 + 1 + 1) = 6 and the most severe score is (3 + 3 + 3 + 3 + 3 + 3) = 18 19. The EQ-TIPS was designed to be amenable to developing preference weights in the future.

### The ages and stages questionnaire (Third Edition) (ASQ)

The ASQ was chosen to test concurrent validity. It is a caregiver-completed questionnaire to monitor development in children aged one month to five years of age 20,21 and is valid and reliable internationally 20,22–24. There are 21 age specific questionnaires each comprising of 30 developmental items which are categorized into five different domains namely: communication, gross motor, fine motor, problem solving and personal social each scored on three levels: yes, sometimes and not yet 20. Answers of yes, sometimes, and not yet are awarded ten, five and zero points respectively. Each dimensions has a total possible score of 60 with a higher score indicating that development is on par with developmental norms. The ASQ has been found to be valid and reliable internationally 20,22–24 and was used to establish concurrent validity of the South African English version of the EQ-TIPS 8.

### FLACC (face, leg, activity, cry, consolability) pain scale

The FLACC Scale is an observational behaviour tool which has been validated in children up to seven years and is used widely in the clinical setting 25–30 and previously used to determine concurrent validity with the South African English version of the EQ-TIPS 8. The scale considers typical pain behaviour in the face, legs, arms, activity, crying and consolability and scores each item from 0-2 with a higher score indicate more pain 25–30.

### Self-designed dietary information questionnaire

Following the methodology followed in the validity testing of the South African English version of the EQ-TIPS 8 the self-designed dietary information questionnaire was used to test the concurrent validity in this study. The child's ability to eat and drink was assessed for the time period ‘today’ for comparison to the dimension included on the EQ-TIPS. The eight items assessed the amount and ability to feed orally with yes/no responses. The total score ranged from 0-8 with a higher score indicating a better eating ability.

All instruments underwent forward-backward translation from English into isiXhosa by two independent translators. The final translated versions of the ASQ was approved by Brookes Publishing.

### Data management and analysis

Age range of the children for inclusion was 0-36 months. To ensure that the instrument was applicable to children across this age band, three age groups were assessed during data analysis and drawn from previous work by Verstraete et al (2020) 8. The age groups were divided according to the child's birthday and included: 0-12 months (≤12 months); 12-24 months (>12 months and ≤24 months) and 24-36 months (>24 months and ≤36 months).

The distribution of frequency of dimension scores across patient care groups (inpatients and out patients was used to determine the feasibility in terms of ceiling and floor effects and missing values. The known group validity was established from the significance of chi-square results of dimension scores and Kruskal-Wallis analysis of the VAS and LSS across groups (inpatient, outpatients and crèche group) and age groups (0-12 months, 12-24 months and 24-36 months). The construct validity of the EQ-TIPS dimensions and the associated domain scores on the ASQ, FLACC scale and Dietary Information was calculated by Spearman's Rho correlation coefficient. Correlation values were interpreted according to Dancey and Reidy guidelines with correlations from 0.1 to 0.3 were considered weak, 0.4 to 0.6 moderate and correlations of 0.7 or above as strong 31. Regression Analysis of the VAS score was used to determine the effect that the dimension scores had on the general health to explore content validity of the EQ-TIPS. Outliers with residual scores >2 Standard Deviations from the mean were excluded for regression analysis. Test-retest reliability was calculated, in a sub-set of crèche children, for dimensions with Cohen's weighted Kappa and intraclass correlation coefficient (ICC) for the VAS and LSS score. Kappa values were interpreted according to Landis and Koch's guidelines with kappa <0.2 poor agreement, 0.21–0.40 fair agreement, 0.41–0.60 moderate agreement, 0.61–0.80 substantial agreement, and kappa >0.81 indicating almost perfect agreement 32. An ICC of >0.7 was considered reliable 33.

The level of statistical significance was set at p≤0.05. Statistical analysis was performed in IBM SPSS Statistics Version 28 and TIBCO Statistical version 13.

## Results

### Descriptive statistics

Research packs were sent to 75 caregivers of crèche children inviting them to participate in the study. Caregivers of 62 children consented and returned the research packs at baseline. The baseline data from seven participants was excluded as less than 50% of the measures were completed. Thus, baseline data from 55 crèche children was included for analysis. A second copy of the EQ-TIPS was sent to the 62 children attending crèche of which 52 returned completed second measures and were included for test-retest analysis.

Seventy-one caregivers of children attending the inpatient hospital facility were eligible for inclusion of which 64 provided informed consent to participate. One participant withdrew due to time constraints thus, responses from 63 inpatient participants were included for analysis. Similarly, 77 caregivers of children attending the outpatient hospital facility were eligible for inclusion of which 69 provided informed consent to participate. Six participants unfortunately withdrew due to attendance of multiple appointments and/or time constraints for completion of the research packs. The responses of 63 outpatient participants were included for analysis.

Most caregivers across health groups were mothers, however there was a significantly higher proportion of grandmother's caring for children from the crèche group than receiving hospital care (X^2^=18.38, p=0.005) (Table 1). Neither the education level (X^2^=5.14, p=0.526) nor the socio-economic status (assessed by receipt of government social grants) (X^2^=2.01, p=0.366) of the caregivers were significantly different between health groups. The age of the children was however significantly associated with health group with most children in the inpatient group being in the 0–12-month group (57%), compared to 12-24 months for the outpatient group (48%) and 24-36 months for the crèche group (65%) (X^2^=50.62, p<0.001).

**Table 1:** Descriptive statistics of the sample

		In patient (n=63)	Out patient (n=63)	Crèche group (n=55)	Total (n=181)	X^2^ (p-value)
		N (%)	N (%)	N (%)	N (%)	
**Proxy Respondents**						
**Relationship of caregiver to child**	Mother	56 (89)	58 (92)	43 (78)	157 (87)	**18.38 (p=0.005)**
	Grandmother	4 (6)	0	10 (18)	14 (8)	
	Other	3 (5)	2 (3)	1 (2)	6 (3)	
	Grandfather	0	3 (5)	1 (2)	4 (2)	
**Caregiver Level of education**	Primary School	5 (8)	11 (17)	4 (7)	20 (11)	5.14 (p=0.526)
	High School	50 (79)	43 (68)	35 (64)	128 (71)	
	Tertiary Education	8 (13)	8 (13)	10 (18)	26 (14)	
	Unspecified	0	1 (2)	6 (11)	7 (4)	
**Social Grant recipient**	Yes	31 (49)	38 (60)	33 (60)	102 (56)	2.01 (p=0.366)
**Child**						
**Gender of Child**	Female	27 (43)	31 (49)	23 (42)	81 (45)	0.79 (p=0.674)
**Age Group of the Child**	0-12 months	36 (57)	16 (25)	6 (11)	58 (32)	**50.62 (p<0.001)**
	12-24 months	18 (29)	30 (48)	13 (24)	61 (34)	
	24-36 months	9 (14)	17 (27)	36 (65)	62 (34)	
**Age (months)**	Median (IQR)	11 (6,21)	19 (12,25)	30 (21,35)	19 (10,29)	**KW-H = 42.9 (p<0.001)**
**Primary Diagnosis of the Child**	None	0	0	52 (95)	52 (29)	
	Unknown	22 (35)	19 (30)	0	41 (23)	
	Other	5 (8)	10 (16)	1 (2)	16 (9)	
	Burns	12 (19)	3 (5)	0	15 (8)	
	Respiratory	5 (8)	9 (14)	1 (2)	15 (8)	
	General Surgery	4 (6)	7 (11)	0	11 (6)	
	Neurodevelopmental	4 (6)	5 (8)	0	9 (5)	
	Cardiac	6 (10)	1 (2)	0	7 (4)	
	Feeding difficulties	1 (2)	3 (5)	0	4 (2)	
	HIV	0 (0)	4 (6)	0	4 (2)	
	Trauma	2 (3)	1 (2)	0	3 (2)	
	Diarrhoea	2 (3)	0	0	2 (1)	
	Eczema	0	1 (2)	1 (2)	2 (1)	

*Bold indicates significance*.

The children had a wide range of conditions where children were most frequently receiving inpatient surgical care for general surgery, following a burn injury or cardiac surgery. Children frequently attended outpatient care for assessment for respiratory illness, assessment pre- or post- general surgery, and assessment and/or treatment for neurodevelopmental concerns (including cerebral palsy, developmental delay, and epilepsy). Notably, 30% of caregivers of children receiving inpatient care and 35% of caregivers of children did not know what the health concerns of their child was. Children from the crèche group were generally healthy.

### Performance of EQ-TIPS across condition groups

There was one missing response for the dimension of movement on the EQ-TIPS and 13 missing VAS responses. Of the missing responses, 7 (50%) were from caregivers of children from the crèche group.

When dimension scores and ceiling effect were compared across health groups, there was significant difference for the dimension of pain and reporting of 111111 (Table 1). Post-hoc analysis showed that there was a significantly higher report of pain for inpatient compared to outpatient (X^2^=7.82, p=0.020) and crèche children (X^2^=35.5, p<0.001) and similarly a higher report of pain in the outpatient group when compared to crèche children (X^2^=15.89, p<0.001). When considering the ceiling effect (111111) there was significantly higher report in the crèche group compared to inpatient (X^2^=23.44, p<0.001) and outpatient (X2=12.40, p<0.001) but no difference between inpatient and outpatient groups (X^2^=2.15, p=0.142).

Comparison of responses across age groups showed a significant difference for the dimensions of pain, communication and reporting of 111111 (Table 1). Post-hoc analysis showed that there was significantly lower report of pain in the 24–36-month group compared to the 0–12-month group (X^2^=16.45, p<0.001) and the 12–24-month group (X^2^=12.41, p=0.002). There was no significant difference between the 0-12 month and 12–24-month groups (X^2^=0.41, p=0.813). The 12–24-month group had a significantly higher report of problems with communication when compared to the 0–12-month group (X^2^=7.36, p=0.025). There were no significant differences for the reporting of problems with communication between the other age groups. The reporting of ceiling effects was higher in the 24–36-month group compared to 0-12 months (X^2^=11.95, p<0.001) and 12-24 months (X^2^=6.88, p=0.008). There was no difference in reporting between 0-12 and 12-24 months (X^2^=0.81, p=0.370).

**Figure 1:**
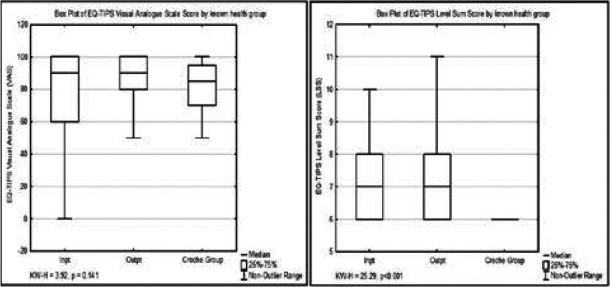
Box plot of EQ-TIPS visual analogue scale scores and level sum scores across known health groups Visual Analogue Scale (VAS) is scored from 0-100 with a higher score indicating a better general health. Level Sum Score (LSS) is scored from 6-18 with a higher score indicating more problems on the EQ-TIPS descriptive system.

**Figure 2:**
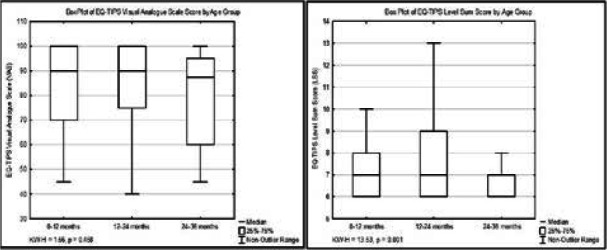
Box plot of EQ-TIPS visual analogue scale scores and level sum scores across age groups Visual Analogue Scale (VAS) is scored from 0-100 with a higher score indicating a better general health. Level Sum Score (LSS) is scored from 6-18 with a higher score indicating more problems on the EQ-TIPS descriptive system.

There was no difference in reporting of EQ-TIPS VAS scores between health groups (KW-H=3.92, p=0.141) (Fig. 1) or age groups (KW-H=1.56, p=0.458) (Fig. 2). The EQ-TIPS LSS showed significant differences between both health groups (Fig. 1) and age groups (Fig. 2). Post-hoc analysis showed that outpatient reported a significantly higher LSS (indicating worse HRQoL) than the crèche group (KW=-30.03, p<0.001) but not compared to inpatient (KW=-14.43, p=0.286). Similarly, inpatients had a higher LSS than the crèche group (KW=-44.46, p<0.001).

There were similar patterns observed with post-hoc age group analysis in that 0-12 months had a significantly higher LSS than the 24–36-month group (KW=28.53, p<0.004) but not compared to the 12–24-month group (KW=1.089, p=1.00). Those aged 0-12 months had a higher report of problems than those aged 24-36 months (KW=28.52, p=0.004).

### Concurrent validity of the EQ-TIPS dimensions

Across all health and age groups there was missing data on the ASQ and Dietary Information which did not allow for scoring, these were excluded for analysis. Table 3 indicates that there was moderate significant agreement between the EQ-TIPS dimension of pain and the FLACC scale. The other dimensions that were hypothesised to correlate all showed weak but significant agreement.

**Table 3:** Spearman's Rho Correlations of EQ-TIPS Dimensions

EQ-TIPS Dimensions	ASQ Gross Motor	ASQ Fine Motor	ASQ Problem Solving	FLACC	ASQ Personal Social	ASQ Communication	Dietary Info
	(n=176)	(n=174)	(n=175)	(n=181)	(n=176)	(n=177)	(n=161)
**Movement**	-0.27**	-0.22**	-0.27**	0.10	-0.21**	-0.14	-0.25**
**Play**	-0.24**	-0.30**	-0.31**	0.13	-0.29**	-0.26**	-0.36**
**Pain**	-0.18*	-0.17*	-0.21**	**0.43** **	-0.17	-0.27**	-0.36**
**Relationships**	-0.25**	-0.19*	-0.25**	0.13	-0.24**	-0.29**	-0.30**
**Communication**	-0.29**	-0.23**	-0.27**	0.07	-0.26**	-0.25**	-0.23**
**Eating**	-0.23**	-0.27**	-0.31**	0.33**	-0.22**	-0.27**	-0.37**

*N=181*

* *p<0.05*

** *p<0.001*

*Shaded cells indicate dimensions and items that were hypothesised to correlate. Bold indicates moderate agreement >0.40. ASQ Ages and Stages Questionnaire*

### Performance of the EQ-TIPS General Health Question measured on the VAS

Multiple regression analysis with the VAS as dependent variable and dummy variables representing the different levels of the dimensions accounted for 4% of the variance. The model improved slightly and accounted for 5% of the variance once nine outliers, with residual scores > 2 Standard Deviations from the mean, were excluded. None of the coefficients significantly detracted from the VAS score. All dimensions detracted from the VAS score except Movement 2, Pain 2, Communication 2 and Communication 3 which marginally increased the VAS score (Table 4).

**Table 4:** Regression Analysis of the EQ-TIPS VAS Score and Dimension Scores

	b*	Std. Err. Of b*	b	Std. Err. Of b	t (143)	p-value
Intercept			96.37	7.82	12.32	0.00
Movement 2	0.14	0.11	7.49	6.16	1.22	0.23
Movement 3	-0.08	0.13	-3.24	4.88	-0.66	0.51
Play 2	-0.09	0.13	-5.87	8.51	-0.69	0.49
Play 3	-0.04	0.17	-1.91	7.93	-0.24	0.81
Pain 2	0.14	0.11	5.56	4.45	1.25	0.21
Pain 3	-0.18	0.11	-5.55	3.37	-1.64	0.10
Relationships 2	-0.19	0.12	-12.12	8.09	-1.50	0.14
Relationships 3	-0.04	0.12	-1.66	5.16	-0.32	0.75
Communication 2	0.02	0.12	1.60	8.73	0.18	0.86
Communication 3	0.05	0.15	2.17	6.26	0.35	0.73
Eating 2	-0.12	0.09	-5.40	3.75	-1.44	0.15
Eating 3	-0.03	0.11	-3.07	10.90	-0.28	0.78

*N=163. 1 No problem, 2 Some problems, 3 A lot of Problems. Adjusted R^2^=0.05 (n=163)*.

b* denotes standardized Beta regression coefficient's; b denotes non-standardized Beta regression coefficients.

### Test-retest reliability

Fifty-two caregivers of school children completed a second copy of the EQ-TIPS to determine test-retest reliability. Six of these children were however reported to have a change in health, due to illness, from the previous completion and were excluded from analysis. Thus, the data of 46 children was assessed for test-retest reliability.

Table 5 indicates that there was fair to moderate and significant agreement for dimensions of movement, play, pain and eating for the EQ-TIPS. across all EQ-TIPS dimensions and the LSS. The VAS was significant and reliable with an ICC=0.72

**Table 5:** Test-retest reliability of the EQ-TIPS dimension and scores

			p-value
Movement	*k*	0.54	**<0.001**
Play	*k*	0.37	**0.011**
Pain	*k*	0.20	**0.009**
Relationships	*k*	-0.04	0.618
Communication	*k*	-0.04	0.668
Eating	*k*	0.29	**0.034**
Level Sum Score	ICC	-0.10	0.624
Visual Analogue Scale	ICC	0.72	**<0.001**

*N=46. k = Cohen's Weighted Kappa, ICC = Intra-Class Correlation Coefficient*.

*Bolded scores indicate significance*.

## Discussion

An experimental isiXhosa version of the EQ-TIPS was developed for use in South Africa. The results of this research suggest that it is feasible, valid and reliable for use in South Africa with some caveats around the performance and understanding of general health and the VAS. The children and caregivers recruited from the crèches in this study were significantly different to the group of children recruited from the hospital facility. The crèche group was older and included more caregivers who were not mothers, but grandmothers. Although there was no significant difference in education level between the groups there was a larger number of caregivers who did not specify education level in the crèche group. Furthermore, seven participants from the crèche group needed to be excluded due to high missingness of data across all measures. When considering missing responses on the EQ-TIPS the majority (50%) were attributed to caregivers from the crèche group. Independent completion may have been difficult for these caregivers with previous validation of the EQ-5D in isiXhosa making use of interviewers 34. Although the questionnaires were not interviewer administered in the hospital group there was a researcher available for questions or clarification.

There was only one missing response on the EQ-TIPS descriptive system indicating that generally caregivers could easily provide data regarding their child's HRQoL. The ceiling effect of each dimension (reporting of no problems) was higher across the inpatient and outpatient groups compared to those previously reported in children with a health condition 8. Reassuringly the ceiling effect across dimensions (111111) was significantly different between those with and without a health condition 11. In keeping with findings from previous studies the report of problems in infants and toddlers with a health condition was highest for the dimensions of pain and eating 8,11. The EQ-TIPS dimensions performed well with no floor effects for children receiving inpatient or outpatient care. As the instrument only has three levels of report, floor effects in children with a health condition would be problematic and would have indicated that the levels were not adequately responsive to health condition.

Known group validity was determined by comparing dimension scores, general health VAS scores and the LSS score between the inpatientt, outpatient and crèche groups and across age groups. At a dimension level pain was significantly different across all three health groups. This was similarly reported in a group of acutely and chronically ill children completing the English version of the EQ-TIPS 8 with differences being more apparent when children with and without a health condition were compared 8,11. Although not significant, there was a higher report of problems with relationships, communication and eating in those receiving inpatient or outpatient hospital care compared to the crèche group. At a composite level (LSS) the EQ-TIPS was able to differentiate between those receiving inpatient and outpatient care and the crèche group, in keeping with results in an older group of children 11. Considering the heterogenous mix of diagnosis across those receiving inpatient and outpatient care the reporting of problems across the other dimensions was anticipated to be mixed 8,11.

There was surprisingly no difference in reporting of general health across health groups as this has been well established in similar research setting on the EQ-TIPS 8,11 and EQ-5D-Y 35–37 which includes the same VAS. If one considers the missingness of VAS data (7%) this could be attributed to a poor understanding of the VAS or the term health. The term health was reported to be difficult to translate and understand in other Bantu languages 38. Although this was not reported in the cognitive debriefing of the translated version of the EQ-TIPS it was only done with ten caregivers and warrants further exploration. This could however also be attributed to the caregivers poor understanding and/or knowledge of their child's health condition if one considers the high report of ‘unknown’ health conditions reported. This could possibly indicate a low rate of health literacy in this group of respondents. Although most caregivers have reported completing high school education, Dowse, Lecoko and Ehlers (2010) report that years of formal schooling was not a good indicator of functional health literacy in isiX-hosa respondents 39. It is recommended that future studies explore the understanding of general health and the VAS regarding infants and toddlers in a similar group of respondents.

Due to the rapid development that takes place in the infant and toddler phase, measures functioning such as the Bayley Scales of Infant Development 40 and Ages and Stages Questionnaire 41 have a number of different items or questionnaires depending on the age of the child. Thus, it was important to establish whether the dimensions included on the EQ-TIPS could measure HRQoL across the ages from 0-36 months. This was difficult to establish in this study due to the age-related differences in each of the health groups, with the majority of children aged 0-12 month receiving inpt care, 12-24 months receiving outpt care and 24-36 months attending a crèche. Due to the heterogenous mix of health conditions reported across the inpt and outpt groups the authors cannot ascertain whether this is an expected finding for health care usage in this age group. The differences in dimension performance for the dimension of pain and the ceiling effect can be directly attributed to the difference in reporting of problems associated with the health groups. The significantly lower report of problems with communication in the 0–12-month group compared to the 12–24-month group may also be associated with health group as this difference was not significant between 0-12 and 24-36 months. This however requires additional research which should ideally include children with conditions known to affect communication.

These results establish concurrent validity of the EQ-TIPS as dimension scores showed weak to moderate significant relationships with standardized measures in comparable domains. The result were similar to those previously reported for the EQ-TIPS 8. The comparison of the EQ-TIPS and ASQ results do however highlight the interdependence of motor, cognitive, emotional and social development in this young age group 42,^43^ with the ASQ dimensions of Gros motor, fine motor, problem solving and personal social showing weak significant correlations across all EQ-TIPS dimensions.

The results of the content validity further suggest a poor understanding of the perceived general health state of these young children on the VAS as the EQ-TIPS dimensions only accounted for 5% of the variance in the VAS score. Previous testing of the English EQ-TIPS in the same research setting, showed that the dimensions accounted for 45% of the variance in the VAS score. This could however also relate to the different value that isiXhosa speaking individuals place on health with a study in adults showing that there was great value placed on environmental factors affecting HRQoL ^44^. This research further highlighted that Urban isiXhosa people placed different value on HRQoL domains with their top ranked domains including food availability, formal housing, family safety and access to medical services ^44^. As these are universal issues and not dependent on age it is postulated that caregivers may consider these important for themselves and their family unit, this however warrants further research.

Test-retest reliability was established for dimensions of movement, play, pain and eating in a sub-group of crèche children. The reasoning for change in reporting of problems at a dimension level was not recorded in this study and may be useful to include in future work. Although the dimensions of communication and relationships showed significant correlations with corresponding ASQ items they did have the weakest relationship in this study and when tested in English for South Africa 8. This may be due to a true change in these dimensions which have conceptual and developmental overlap 7 but warrants further investigation. It is further recommended that future studies that plan to establish the test-retest reliability include children with health conditions known to be stable to ensure greater variation of reporting of problems in each of the dimensions.

## Conclusion

This research indicates that the isiXhosa EQ-TIPS is a valid and reliable measure of HRQoL for very young children in South African and we suggest that it be included in the assessment of children with health conditions within this context. Further research is needed to establish whether there are age related differences across the EQ-TIPS dimensions and whether the dimensions are responsive to a change in health condition. Research on the understanding of the VAS and the perceived general health of infants and toddlers is warranted. This will ensure better conceptualisation of the EQ-TIPS and the expectation and understanding of health and health care in South Africa by isiXhosa speaking people.

## Figures and Tables

**Table 2:** Dimension scores of the EQ-TIPS across known health and age groups

		Total (n=181)	Inpt (n=63)	Outpt (n=63)	Crèche Group (n=55)	Health groups	0-12 months (n=58)	12-24 months (n=61)	24-36 months (n=62)	Age groups
		N (%)	N (%)	N (%)	N (%)	X2 (p-value)	N (%)	N (%)	N (%)	X2 (p-value)
Movement	1	153 (85)	51 (81)	53 (84)	49 (89)	1.67 (p=0.80)	49 (84)	50 (82)	54 (87)	6.60 (p=0.158)
	2	22 (12)	10 (16)	7 (11)	5 (9)		5 (9)	9 (15)	8 (13)	
	3	5 (3)	2 (3)	2 (3)	1 (2)		4 (7)	1 (2)	0	
Play	1	165 (91)	55 (87)	58 (92)	52 (95)	2.57 (p=0.633)	53 (91)	53 (87)	59 (95)	4.75 (p=0.314)
	2	13 (7)	7 (11)	4 (6)	2 (4)		3 (5)	7 (11)	3 (5)	
	3	3 (2)	1 (2)	1 (2)	1 (2)		2 (3)	1 (2)	0	
Pain	1	120 (66)	27 (43)	41 (65)	52 (95)	**37.21 (p<0.001)**	31 (53)	36 (59)	53 (85)	**17.32 (p=0.002)**
	2	50 (28)	28 (44)	20 (32)	2 (4)		21 (36)	20 (33)	9 (15)	
	3	11 (6)	8 (13)	2 (3)	1 (2)		6 (10)	5 (8)	0	
Relationships	1	161 (89)	55 (87)	53 (84)	53 (96)	4.44 (p-0.350)	54 (93)	50 (82)	57 (92)	7.34 (p=0.119)
	2	14 (8)	6 (10)	7 (11)	1 (2)		3 (5)	7 (11)	4 (6)	
	3	5 (3)	2 (3)	2 (3)	1 (2)		1 (2)	4 (7)	0	
Communication	1	166 (92)	56 (89)	57 (90)	53 (96)	2.56 (p=0.634)	56 (97)	51 (84)	59 (95)	**10.67 (p=0.031)**
	2	10 (6)	5 (8)	4 (6)	1 (2)		0	7 (11)	3 (5)	
	3	5 (3)	2 (3)	2 (3)	1 (2)		2 (3)	3 (5)	0	
Eating	1	139 (77)	46 (73)	45 (71)	48 (87)	5.12 (p=0.276)	43 (74)	42 (69)	54 (87)	6.15 (p=0.188)
	2	36 (20)	15 (24)	15 (24)	6 (11)		13 (22)	16 (26)	7 (11)	
	3	6 (3)	2 (3)	3 (5)	1 (2)		2 (3)	3 (5)	1 (2)	
111111		90 (50)	20 (32)	28 (44)	42 (76)	**24.46 (p<0.001)**	21 (36)	27 (44)	42 (68)	**13.02 (p<0.001)**

## Data Availability

The datasets used and/or analysed during the current study are available from the corresponding author on reasonable request. The EQ-TIPS is an experimental version of the EQ-5D-Y Proxy, permission for use needs to be made with the EuroQoL Research Foundation.
